# Differences in antigen-specific CD4+ responses to opportunistic infections in HIV infection

**DOI:** 10.1002/iid3.50

**Published:** 2015-04-29

**Authors:** Katrina M Pollock, Damien J Montamat-Sicotte, Graham S Cooke, Moses S Kapembwa, Onn M Kon, Lisa Grass, Robert D Sampson, Graham P Taylor, Ajit Lalvani

**Affiliations:** 1 Tuberculosis Research Centre Department of Respiratory Medicine, National Heart and Lung Institute, Imperial College LondonLondon, UK; 2 Section of Infectious Diseases, Department of Medicine, Imperial College LondonLondon, UK; 3 Department of GU and HIV Medicine, The North West London Hospitals NHS TrustLondon, UK; 4 Tuberculosis Service, St Mary's Hospital, Imperial College Healthcare NHS TrustLondon, UK; 5 Centre for Respiratory Infection, Flow Cytometry Facility, National Heart and Lung Institute, Imperial College LondonLondon, UK

**Keywords:** *Candida albicans*, cytomegalovirus, Epstein-Barr virus, HIV, *Mycobacterium tuberculosis*

## Abstract

HIV-infected individuals with severe immunodeficiency are at risk of opportunistic infection (OI). Tuberculosis (TB) may occur without substantial immune suppression suggesting an early and sustained adverse impact of HIV on *Mycobacterium tuberculosis* (MTB)-specific cell mediated immunity (CMI). This prospective observational cohort study aimed to observe differences in OI-specific and MTB-specific CMI that might underlie this. Using polychromatic flow cytometry, we compared CD4+ responses to MTB, cytomegalovirus (CMV), Epstein-Barr virus (EBV) and *Candida albicans* in individuals with and without HIV infection. MTB-specific CD4+ T-cells were more polyfunctional than virus specific (CMV/EBV) CD4+ T-cells which predominantly secreted IFN-gamma (IFN-γ) only. There was a reduced frequency of IFN-γ and IL-2 (IL-2)-dual-MTB-specific cells in HIV-infected individuals, which was not apparent for the other pathogens. MTB-specific cells were less differentiated especially compared with CMV-specific cells. CD127 expression was relatively less frequent on MTB-specific cells in HIV co-infection. MTB-specific CD4+ T-cells PD-1 expression was infrequent in contrast to EBV-specific CD4+ T-cells. The variation in the inherent quality of these CD4+ T-cell responses and impact of HIV co-infection may contribute to the timing of co-infectious diseases in HIV infection.

## Introduction

*Mycobacterium tuberculosis* (MTB) is a common cause of HIV-related opportunistic infection (OI) in endemic 1 and non-endemic areas 2. Tuberculosis (TB) occurs more frequently compared with HIV-uninfected individuals in early 3,4 treated 5 and advanced HIV infection. By comparison, common OIs e.g. cytomegalovirus (CMV), Epstein-Barr virus (EBV), and *Candida albicans* (*C. albicans*) cause disease at defined later stages of CD4 cell depletion 6–10. Highly active antiretroviral therapy (HAART) significantly reduces disease caused by *C. albicans*, EBV and CMV but may be less effective in protecting against an active TB infection. HAART significantly reduced TB incidence in one study, although cases still occurred in those who received HIV therapy 11. Similar results in other cohorts suggest that whilst incidence is reduced, MTB-specific immunity may not completely regenerate with HAART and TB incidence is not restored to background levels 12. These observations of the timing of different co-infections may reflect a unique effect of HIV co-infection on MTB-specific immunity compared with other OIs that occurs even in those with treated HIV infection.

Host responses to MTB, CMV, EBV and *C. albicans* share a dependence on cell-mediated immunity (CMI), in particular antigen-specific CD4+ and CD8+ T-cell responses. The differential impact of HIV co-infection may lie in its effects on T-cell immunity; for example, some pathogen-specific CD4+ T-cell subsets may be more vulnerable to functional modulation or destruction by HIV infection than others and some may be less able to regenerate during immune restoration with HAART.

We hypothesised that in HIV co-infection MTB-specific CMI would differ in frequency and phenotype from CMI specific for other OIs. We compared pathogen-specific responses to MTB, CMV, EBV and *C. albicans* in individuals with and without HIV infection. Secondly, we compared the differential impact of HIV infection on the pathogen-specific responses within the same individuals at the same time point. Whilst previous studies have focused on T-cells specific for these OIs, none to our knowledge have done so simultaneously with MTB-specific T-cell immunity in HIV co-infected individuals. We used polychromatic flow cytometry to quantify 7 non-overlapping functional CD4+ T-cell subsets defined by IFN-gamma (IFN-γ), IL-2 (IL-2) and TNF-alpha (TNF-α) secretion. We assessed these functional subsets for memory phenotype (CD45RA, CCR7 expression), CD127 loss of expression of which on CD4+ T-cells correlates with HIV disease progression 13 and programmed death-1 (PD-1), a marker of T-cell exhaustion. Expression of these two markers on different OI pathogen-specific T-cells in the context of HIV co-infection has not previously been investigated.

## Results

### MTB-specific CD4+ T-cells are more polyfunctional than CMV-specific T-cells, which predominantly secrete IFN-γ-only

The demographics and HIV-infection clinical parameters of participants are reported in Table1; HIV-infected individuals were in the early or treated stages of infection (60% on HAART) with a median (IQR) CD4 count of 455 cells/µl (356,530) and HIV viral load (VL) of 10 copies/mL (10,12,691). Those on HAART *n* = 6 had a median (IQR) CD4 count of 372 (300,563) cells/µl and median (IQR) VL of 10(10,10) RNA copies/mL i.e. below the threshold of detection. The median (IQR) duration of HAART was 3.4 years (0.8, 8.0). For those not on HAART, (*n* = 4) the median (IQR) CD4 count was 500 (443, 528) cells/µl and median (IQR) VL was 12,904 (3701, 37,621) copies/mL.

**Table 1:** Demographics and test results of participants

	HIV		No HIV	
10	% or IQR	11	% or IQR
Median (IQR) age	37	(24,40)	34	(30,36)
Male	6	(60.0)	4	(36.4)
Female	4	(40.0)	7	(63.6)
Black African	8	(80.0)	6	(54.5)
Asian	0	(0.0)	3	(27.3)
Caucasian/Central S America	2	(20.0)	2	(18.2)
HIV positive	10	(100.0)	0	(0.0)
HIV negative	0	(0.0)	6	(54.5)
HIV untested^a^	0	(0.0)	5	(45.5)
Median (IQR) CD4 cells/µl^b^	455	(356;530)	NA	NA
Median (IQR) CD4%	25	(15,30)	NA	NA
Median (IQR) HIV RNA copies/mL	10	(10;12691)	NA	NA
HAART treated	6	(60.0)	NA	NA
HAART untreated	4	(40.0)	NA	NA
BCG vaccinated	9	(90.0)	8	(72.7)
BCG unvaccinated	0	(0.0)	2	(18.2)
BCG history unknown	1	(10.0)	1	(9.1)
T.Spot +/− TST positive	10^c^	(100.0)	11	(100.0)
Proportion with positive total response to				
PPD	10/10	(100.0)	11/11	(100.0)
EBV infected cell extract	10/10	(100.0)	11/11	(100.0)
CMV infected cell extract	10/10	(100.0)	10/11	(90.9)
Candida albicans	6/6	(100.0)	10/10	(100.0)

IQR = interquartile range.

a Local clinical guidelines at the time did not specify testing for HIV infection in individuals undergoing screening for latent tuberculosis infection, however all those without an HIV test had normal CD4/CD8 ratios in the experimental assay (>1.2).

b CD4 counts according to standardized clinical protocols for patients with HIV infection were not available for HIV-uninfected individuals.

c One individual had received 3 days chemoprophylaxis treatment at the time of recruitment.

**Figure 1:**
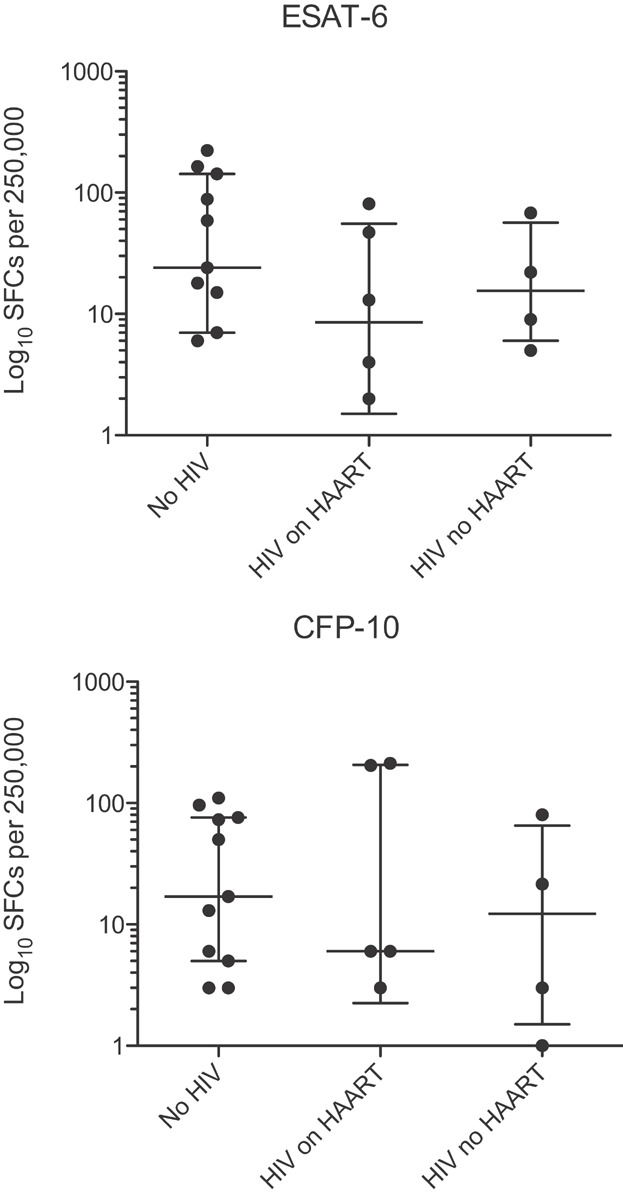
IFN-γ ELISpot responses. Graphs show log_10_ frequency of spot forming cells per 250,000 PBMCs responding to overnight stimulation with MTB-specific antigens, ESAT-6 (top graph) and CFP-10 (bottom graph) in all participants without evidence of HIV infection (left column) with HIV infection on highly active antiretroviral therapy (HAART, middle column) and not on HAART (right column). Kruskal–Wallis test with Dunn's post-test comparison showed no significant difference between the three groups for either antigen. One untreated individual's assay failed at T0 but was positive at T1. Two individuals had a positive response to ESAT-6 and/or CFP-10 in the commercial assay only.

All participants had evidence of MTB sensitisation based on either a clinical ELISpot (T-Spot). TB or responses to the MTB-specific antigens ESAT-6 and CFP-10 measured in a laboratory IFN-γ ELISpot. As shown in Figure 1 there was no difference in frequency of spot forming cells between individuals with or without evidence of HIV infection or by antiretroviral therapy.

**Figure 2:**
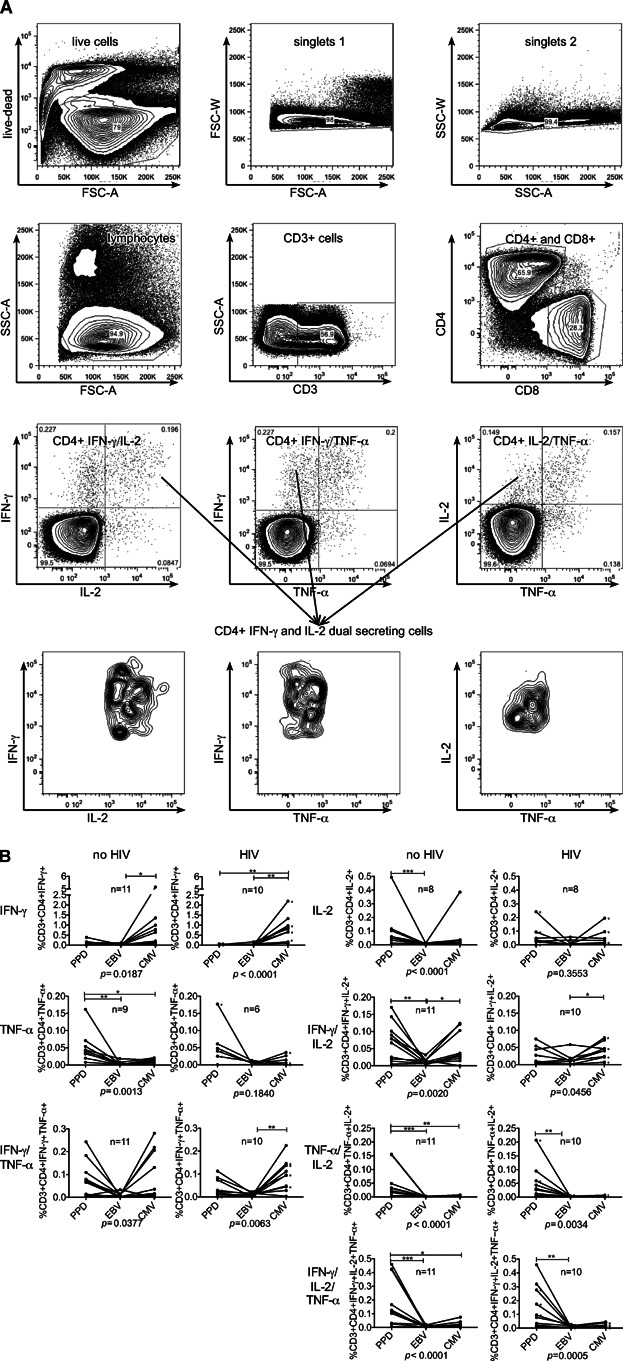
Frequency distribution of functional subset responses in participants with and without HIV co-infection. Representative gating strategy showing results from an individual without evidence of HIV infection. PBMCs were stimulated overnight with PPD and stained for intracellular and phenotypic markers before collection on an LSR II flow cytometer. Data were gated on live cells, singlets, lymphocytes, CD3+ cells and then CD4+ and CD8+ cells. CD4+ cells were then gated for expression of IFN-γ, IL-2, and TNF-α and Boolean gating used to create 7 non-overlapping subsets for example IFN-γ and IL-2 dual producing CD4+ cells (A). Graphs show frequency of all 7 CD4+ cytokine subsets responding to PPD, EBV and CMV in those with and without evidence of HIV infection. Those with HIV infection not on HAART are marked with an asterisk (B). Each line represents a comparison of the response to multiple pathogens within a single individual. Analysis using Friedman test and Dunn's Multiple Comparison test: *P < 0.05, **P < 0.01, ***P < 0.001. Analysis shows all those with a positive response to at least one of the three antigens, non-responders to all three antigens were excluded. Experiments were conducted in *n* = 10 HIV, *n* = 11 non-HIV. All individuals had a positive response in each cytokine subset to at least one antigen except in the following cases; for the HIV group CD4+ IL-2-only-secreting cells *n* = 8 and CD4+ TNF-α-only secreting cells *n* = 6 had a positive response and for the non-HIV group CD4+ IL-2-only-secreting cells *n* = 8 and CD4+ TNF-α-only secreting cells *n* = 9 had a positive response. When compared with those with HIV infection, those without HIV infection had relatively more frequent PPD-specific cells secreting IFN-γ and IL-2 (*P* = 0.015).

Pathogen-specific CD4+ responses to PPD, EBV and CMV were compared using Boolean gating to create 7 non-overlapping CD4+ cell subsets (Fig. 2A). All participants had a CD4+ T-cell response to PPD and EBV and all but one to CMV in at least one functional cytokine subset. In general the EBV-specific response was low frequency and monofunctional, the CMV-specific response was high frequency and monofunctional and the MTB-specific response was intermediate frequency and polyfunctional. PPD induced a higher frequency of CD4+ cells secreting TNF-α-alone, with IL-2 and with IFN-γ and IL-2 compared with EBV and CMV in those without evidence of HIV infection, with similar trends in those with HIV infection (Fig. 2B).

CMV induced a higher frequency of IFN-γ-only secreting CD4+ cells than EBV in those without evidence of HIV infection and a higher frequency than MTB- and EBV-specific IFN-γ-only CD4+ cells in those with HIV infection (Fig. 2B). A principal impact of HIV was to attenuate the frequency of the MTB-specific IFN-γ and IL-2-dual response but not other pathogen-specific IFN-γ and IL-2-dual responses (Fig. 2B). When compared with those with HIV infection, those without evidence of HIV infection had relatively more frequent CD4+ MTB-specific cells secreting IFN-γ and IL-2 (*P* = 0.015).

### MTB-specific responses derive mostly from the central memory T-cell pool whereas responses to other OIs are more differentiated

**Figure 3:**
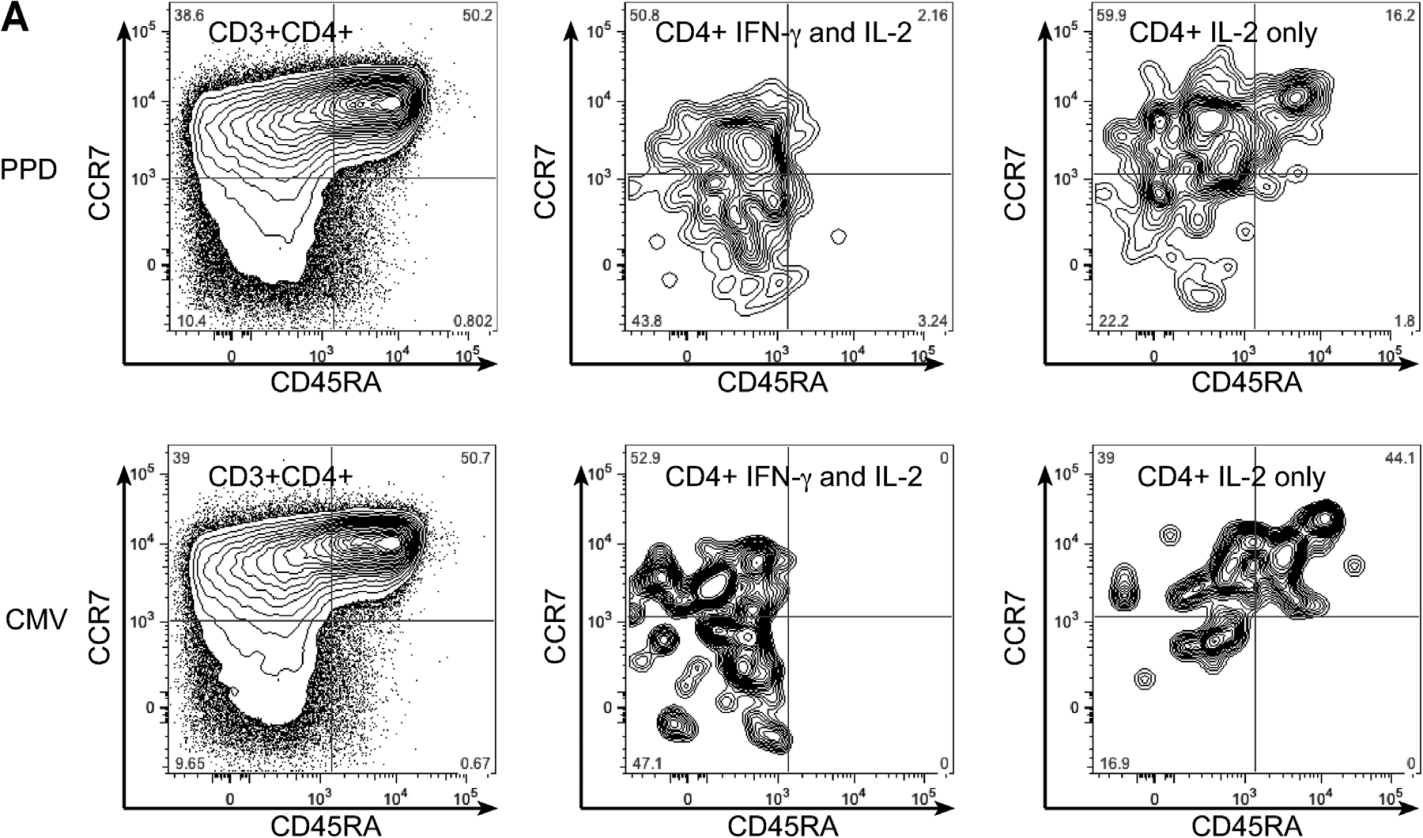
Comparison of memory phenotype in each CD4+ functional subset: Representative gating stategy for one individual without HIV infection. Contour plots show CCR7 and CD45RA expression of from left to right bulk CD4+ cells, IFN-γ and IL-2 dual- and IL-2 only secreting CD4+ cells in response to overnight stimulation with PPD (top row) and CMV (bottom row) (A). Graphs show from left to right, proportion of cells that were naïve (CD45RA+CCR7+), *T*_CM_ (CD45RA−CCR7+), *T*_EM_ (CD45RA−CCR7−) and *T*_EMRA_ (CD45RA+CCR7−) in each CD4+ functional subset responding to EBV, CMV, *C. albicans* and PPD in all participants. Graphs show CD4+ cells secreting IFN-γ alone (B), IFN-γ and TNF-α (C) and those secreting IFN-γ and IL-2 (D). Graphs show only those with a positive response in each functional subset (number indicated above graph). p values show Friedman test with Dunn's Multiple Comparison test; *P < 0.05, **P < 0.01, ***P < 0.001.

We next examined the phenotype of CD4+ pathogen-specific T-cells according to whether they were naïve (CD45RA+CCR7+), central memory; *T*_CM_ (CD45RA−CCR7+), effector memory; T_EM_ (CD45RA−CCR7−) or CD45RA+ effector memory; T_EMRA_ (CD45RA+CCR7−) (Fig. 3A). MTB-specific CD4+ functional effector cells secreting IFN-γ and TNF-α were enriched in the *T*_CM_ subset whereas responses to EBV, CMV and for the most part *C. albicans* were more differentiated. This was striking for IFN-γ-only secreting CD4+ cells responding to CMV and *C. albicans*, which were mostly *T*_EM_ (Fig. 3B). Similarly CD4+ cells secreting IFN-γ and TNF-α were enriched in the *T*_EM_ subset for CMV, EBV and *C. albicans* responses but not for PPD, which were enriched in the *T*_CM_ subset (Fig. 3C). These CMV-specific CD4+ cells were more differentiated in HIV co-infection than PPD and/or EBV (supplementary Fig. 1). CD4+ cells secreting IL-2 with or without other cytokines tended to derive from the *T*_CM_ subset irrespective of pathogen specificity for example IFN-γ and IL-2-dual secreting CD4+ cells (Fig. 3D). The presence of HIV co-infection did not affect the proportion of antigen-specific cells that were *T*_CM_ for any pathogen-specificity (data not shown).

### CD127 expression was greatest on MTB-specific cells

**Figure 4:**
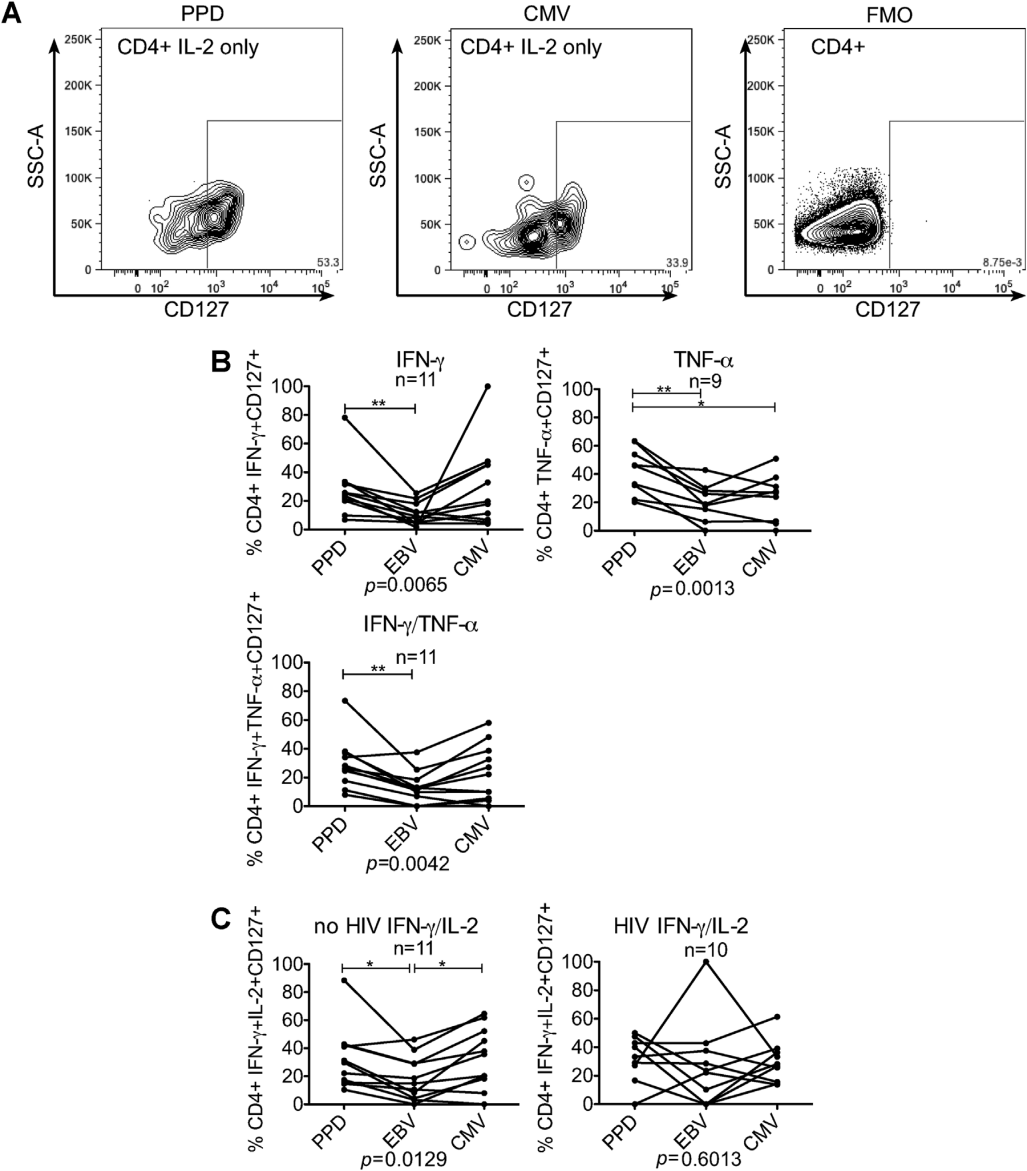
Comparison of CD4+ functional subsets that were CD127+: Representative gating strategy, using fluorescence minus one (FMO) control to gate on CD127+ CD4+ cells from each Boolean gated subset. CD4+ cells secreting IL-2 only in response to overnight stimulation with PPD (left) and CMV cell extract (middle) are shown with the FMO (right) (A). Graphs show percentage of cells in each CD4+ functional effector subset that were CD127 positive in those without evidence of HIV co-infection (B). Graphs show percentage of CD4+ cells secreting IFN-γ and IL-2 that expressed CD127 in those without evidence of HIV infection (left column) and with HIV infection (right column) (C). Each line represents a comparison of the response to multiple pathogens within a single individual. Analysis using Friedman test and Dunn's Multiple Comparison test *P < 0.05, **P < 0.01, ***P < 0.001. Analysis shows all those with a positive response to at least one of the three antigens, non-responders were excluded (number indicated above graph).

Antigen-specific CD4+ cells were examined for expression of CD127 (Fig. 4A). MTB-specific CD4+ functional effector cells secreting IFN-γ alone, TNF-α alone or IFN-γ and TNF-α more frequently expressed CD127 compared with EBV and/or CMV-specific cells in those without evidence of HIV co-infection (Fig. 4B). These differences were not seen in HIV co-infection (data not shown). In HIV co-infection CD127 was relatively less frequently expressed on MTB-and CMV-specific IFN-γ and IL-2-dual-secreting cells compared with EBV-specific CD4+ cells than in those without evidence of HIV infection (Fig. 4C).

### PD-1 expression on MTB-specific cells was significantly lower than other OIs

**Figure 5:**
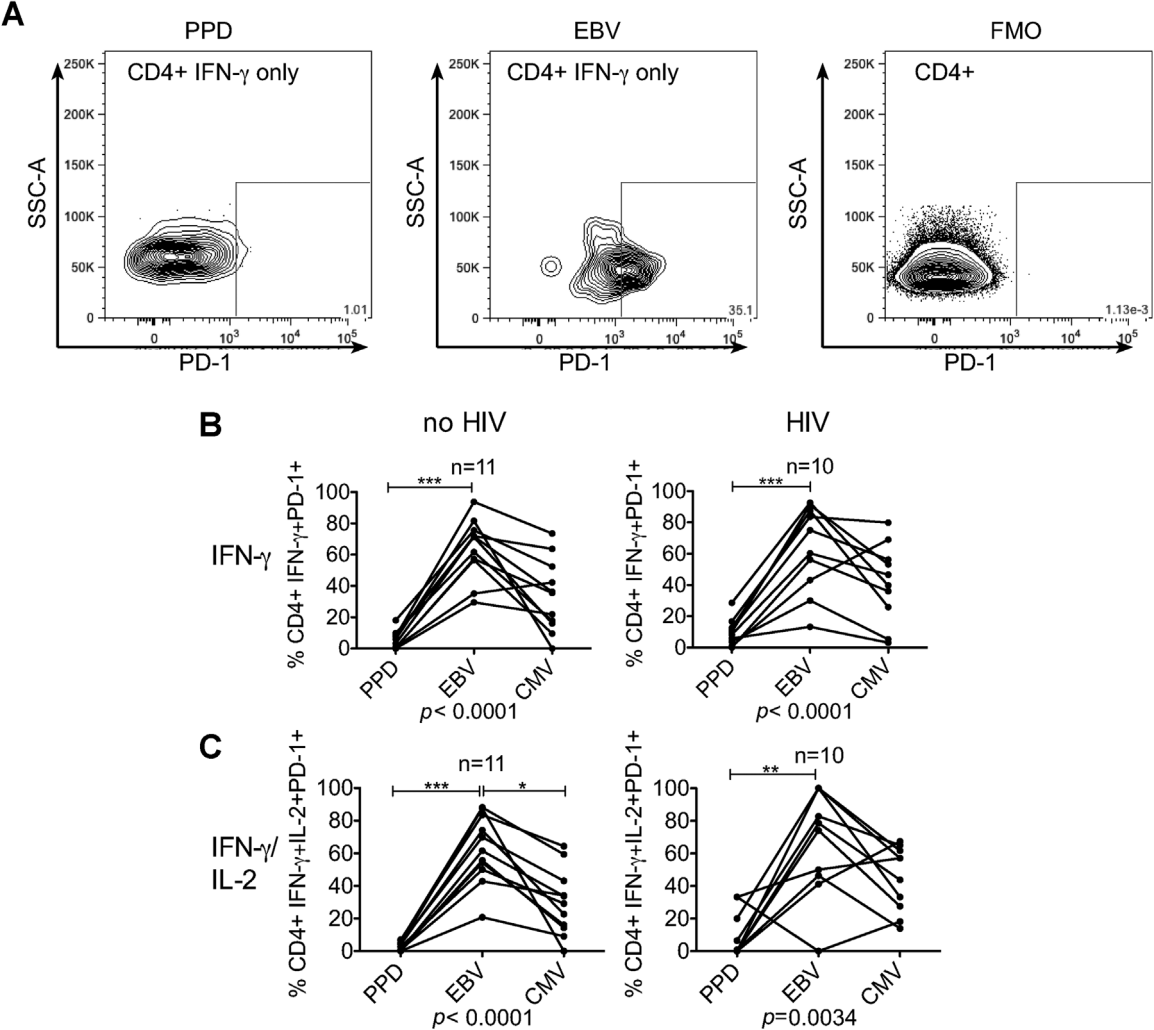
Comparison of percentage of CD4+ pathogen-specific subsets expressing PD-1. Representative gating strategy, using fluorescence minus one (FMO) control to gate on PD-1+ CD4+ cells from each Boolean gated subset. CD4+ cells secreting IFN-γ only in response to overnight stimulation with PPD (left) and EBV cell extract (middle) are shown with the FMO (right) (A). Graphs show frequency of CD4+ cells expressing IFN-γ alone (B) and IFN-γ and IL-2 (C) that were PD-1 positive in participants with and without evidence of HIV infection. Only those with a positive response to any antigen were included (number indicated above graph). Each line represents a comparison of the response to multiple pathogens within a single individual. p values are results of Friedman test and Dunn's Multiple Comparison test *P < 0.05, **P < 0.01, ***P < 0.001.

Antigen-specific cells were next examined for expression of PD-1 (Fig. 5A). Across most subsets especially those secreting IFN-γ, EBV-specific CD4+ cells more frequently expressed PD-1 than MTB-specific CD4+ cells in those with and without evidence of HIV co-infection for example IFN-γ-only secreting cells (Fig. 5B). In contrast to other pathogen-specific CD4+ cells, MTB-specific T-cells infrequently expressed PD-1 across all subsets except IL-2-only secreting cells, where expression of PD-1 tended to be infrequent in response to EBV, CMV and *C. albicans* (Supplementary Fig. 2). CMV-specific cells expressed PD-1 relatively more frequently in those with HIV co-infection in polyfunctional subsets secreting IFN-γ e.g. IFN-γ and IL-2-dual secreting cells (Fig. 5C).

## Discussion

In our cohort of mostly treated or relatively immunocompetent HIV-infected individuals and those without evidence of HIV co-infection, MTB-specific T-cells were more frequently polyfunctional and less differentiated than virus-specific T-cells. CMV and EBV-specific T-cells were more frequently monofunctional (dominated by IFN-γ) and more differentiated than MTB-specific cells. HIV co-infection was associated with an increased frequency of CMV-specific IFN-γ-only and TNF-α-only CD4+ cells compared with PPD. It was also associated with the relative loss of CD127 expression on CD4+ MTB-specific functional effector cells secreting IFN-γ and/or TNF-α.

Compared to other pathogens, MTB-specific T-cells were uniquely impacted by HIV co-infection, as there was attenuation of the frequency of the CD4+ IFN-γ and IL-2-dual response. These cells were principally derived from the *T*_CM_ subset irrespective of pathogen specificity and MTB-specific IFN-γ and IL-2-dual secreting CD4+ cells expressed relatively little CD127 in HIV co-infection and very little PD-1 regardless of HIV infection. Even in individuals with the early or treated stages of HIV infection the incidence of active TB infection is elevated 4,12,14. The incidence of other OIs including EBV, CMV and *C. albicans* are conversely more closely associated with CD4 count decline and HAART may therefore have a relatively more protective effect. Some T-cell subsets may be especially vulnerable to HIV-mediated destruction contingent upon cytokine secretion e.g. IL-2, or pathogen-specificity or a combination of the two. This may be related to the absence of other cytokines such as MIP-1β, binding HIV co-receptors 15–17. Although we did not measure CCR5 expression, the IL-2-secreting polyfunctional MTB-specific CD4+ cell response could be more vulnerable to HIV than pathogen-specific CMI where CD4+ functional effectors predominate e.g. CMV. Immune competence to these OIs may therefore be relatively resilient until CD4 count has significantly declined.

Our finding that MTB-specific IFN-γ and IL-2-dual-secreting cells were significantly less frequent in our HIV co-infected cohort with relatively preserved or reconstituted immunity suggests that these cells may be both especially vulnerable in early HIV infection and fail to reconstitute with HAART. Antigen-specific T-cells secreting IL-2 with or without IFN-γ have been associated with containment (or resolution) of viral infections 18–20 and TB 21–23. The total MTB-specific IFN-γ response protects against the development of active TB in HIV-infected individuals 24 and MTB-specific functional effector and polyfunctional cells were less frequent in the lung of HIV-infected individuals 25. This relationship was not observed for other functional subsets or for other pathogens, pinpointing CD4+ IFN-γ and IL-2-dual-secreting cells as a possible correlate of MTB containment in vivo. The resulting potential for loss of immunoprotection and replication and spread of MTB remains to be explored in animal models or larger longitudinal cohorts.

Expression of CD127 may indicate potential for cellular survival. The IL-7 receptor heterodimer prevents cellular apoptosis through IL-7 signalling 26. T-cell activation causes down-regulation of CD127 27, and relatively low levels of expression were seen on PPD stimulated CD4+ cells although they were predominantly *T*_CM_. In those without HIV co-infection PPD-specific CD4+ cells secreting IFN-γ with or without TNF-α and expressing CD127 were relatively more frequent but this expression was reduced in HIV co-infection indicating that these cells might be relatively short-lived. Furthermore CD4+ PPD-specific cells secreting IFN-γ and IL-2 and expressing CD127 were relatively less frequent in HIV co-infection indicating another potential mechanism of the loss of this key subset.

Our data indicate pathogen-related variance in expression of CD45RA and CCR7 of some subsets analogous to work on CD8+ cells 28,29 and CD4+ cells 30. CD4+ cells secreting IL-2 with or without other cytokines were principally derived from the *T*_CM_ subset irrespective of pathogen specificity. The most striking differences were in IFN-γ-only, TNF-α-only and IFN-γ and TNF-α dual-secreting CD4+ cells where the PPD-specific response was mostly *T*_CM_ and responses to other pathogens especially CMV were more differentiated. There was a dichotomy between the relationship of pathogen specificity, cell phenotype and functionality between cells that secreted IL-2 and those that did not. Whilst linked T-cell functionality and memory phenotype is well established in several models 31, our data suggest a more flexible relationship between surface phenotype and cytokine expression in human HIV and MTB coinfection. As we showed previously, this may be related to antigen burden 32. In HIV co-infection there was enrichment of *T*_EM_ CMV-specific cells secreting IFN-γ, consistent with an HIV-associated subclinical increase in CMV antigen load driving the size and differentiation of the CMV-specific effector-memory cellular immune response.

We observed significant pathogen-specific differences in the frequency of CD4+ PD-1 expressing cells. The PD-1 pathway plays a role in T-cell exhaustion in HIV 33,34 and MTB 35 and may be tissue protective 36. Our data suggest a less significant role for PD-1 on CD4+ MTB-specific T-cells in LTBI where antigen load is low. It is unlikely that our cohort had a high EBV viral load however, which begs a different explanation of the high frequency of CD4+ EBV-specific PD-1+ cells we observed. The frequency of cells expressing PD-1 may be an inherent quality of the pathogen-specific T-cell response that could have a direct relationship to IFN-γ secretion that varies by pathogen. The generally low absolute frequency of EBV-specific CD4+ T-cells but high proportion of PD-1-expressing T-cells within them is notable and in contrast to other pathogens. In those without HIV co-infection a higher percentage of several EBV-specific CD4+ T-cell subsets expressed PD-1 than CMV-specific cells. This suggested that in HIV co-infection these CMV-specific subsets were more likely to express PD-1 again consistent with an HIV-related increase in the CMV immune response.

Our observations bring together detailed comparative analysis of CMI to opportunistic pathogens, and the OI-specific impacts of HIV infection. Our study was limited by the small cohort size, which included a mostly treated HIV-group. Therefore, while we were able to demonstrate significant differences we were unable to prove the absence of weaker associations, which would require a larger cohort. Pathogen-specific differences that we observed in the natural frequency and phenotype of the CD4+ response may underlie inherent immune vulnerabilities in HIV co-infection and shape the timing of opportunistic infections.

## Materials and Methods

### Case selection

Individuals undergoing clinical screening for or with risk factors for latent TB infection e.g. healthcare work with TB patients or history of exposure to TB, were recruited from three London, UK clinical centres from 2008 to 2010. Participants were ≥18 years and provided written informed consent, National Research Ethics Service approval (07/H0712/85). *n* = 21 individuals with and without HIV infection were selected for immunological studies. Evidence of immune sensitisation to MTB was defined as a positive IFN-γ MTB-specific ELIspot (either commercial or laboratory assay) and no evidence of active TB. Those with a previous history of treated TB infection and no re-exposure were excluded. Evidence of HIV infection was determined by serology in a clinical pathology laboratory. The presence of cellular immune responses to the OI pathogen-specific antigens on flow-cytometry (see below) for CMV, EBV or *C. albicans*, was deemed as evidence of immune sensitisation with the respective pathogens. Non-responders were excluded from analysis where indicated.

### IFN-γ ELISpot

Was performed as previously described 37 in singlicate using fresh or frozen peripheral blood mononuclear cells (PBMCs) and cells left unstimulated or stimulated with phytohaemagglutinin (PHA), purified protein derivative (PPD) and pools of MTB-specific 15-mer overlapping peptides including ESAT-6 and CFP-10 (10 µg/mL per peptide final concentration). Positive responders were those with a test well that was greater than or equal to twice the negative control well and at least 5 spot forming cells greater than the negative control well.

### Intracellular cytokine staining

Performed as previously described 32. Briefly, thawed PBMCs were cultured for 16 h (37°C 5% CO_2_) in 10% human serum in Roswell Park Memorial Institute medium (RPMI) at a concentration of 1 × 10^7^ cells/mL. Cells were left unstimulated or stimulated with PMA-Ionomycin, PPD, EBV infected cell extract, CMV infected cell extract or *C. albicans* bulk antigen. After 2 h, monensin (2 μM final concentration) was added. Cells were washed and stained with a dead cell marker (LIVE/DEAD® Fixable Dead Cell Stain Kits, aqua, Invitrogen, Paisley, UK) for 30 min at 4°C in PBS, washed in PBS and placed in FC block buffer (10% human serum in FACS solution) for 20 min at 4°C. Staining was with a pre-titrated and optimised antibody cocktail with fluorochrome-conjugated antibodies against CD3-APC-Alexa Fluor®750, CD4-Qdot®605 and CD45RA- CD4-Qdot®655 (Invitrogen), CD8-APC, CCR7-PE-Cy™7 and CD127-FITC (BD Biosciences) and PD-1-PerCP/Cy5.5 (BioLegend, London UK). The cells were fixed and permeabilised using BD Cytofix/Cytoperm™ Fixation/Permeabilisation kit (BD Biosciences) for 20 min at 4°C. The cells were washed twice with Perm/Wash solution (BD Biosciences, Oxford, UK) then stained with pre-titrated fluorochrome-conjugated antibodies in Perm/Wash solution with IFN-γ-V450, IL-2-PE and TNF-α-AlexaFluor 700 (BD Biosciences) for 30 min 4°C. The cells were acquired straightaway using an LSR-II flow cytometer and 1 × 10^6^ events acquired where possible. Anti-Rat and Anti-Mouse Ig compensation beads (BD Biosciences) were used to set compensation parameters. Fluorescence minus one (FMO) controls were used to set gates.

### Antigens

PPD was obtained from Serum Statens Institute and resuspended in RPMI and used at a final concentration of 16.7 µg/mL. EBV-infected (Human B cell) cell extract, virus strain P3HR1 (EV012 EastCoast Bio, final concentration 10 µg/mL), CMV-infected cell (Normal Human Dermal Fibroblast) extract, virus strain AD169 (CV001 EastCoast Bio, final concentration 10 µg/mL) and *C. albicans* bulk antigen (BA117VS virion\serion, final concentration 20 µg/mL). The reproducibility and acceptability of using these extracts to study cellular EBV and CMV responses has been established 38. In some participants PBMC availability was insufficient for responses to *C. albicans* to be tested.

### Data analysis

The data were analysed on FlowJo version 9.2 FlowJo LLC 385 Williamson Way, Ashland OR 97520 and gated as previously described, briefly dead cells and doublets were excluded, then lymphocytes chosen, cells were gated as CD3+ then CD4+ and then Boolean gating used to create seven non-overlapping subsets e.g. CD4+IFN-γ and IL-2-dual secreting cells 32. All responses were normalised to the unstimulated, fully stained control. Positive responders were defined as those with a response that was ≥2 times the background and with a frequency of >0.001 CD4+ cells. Each subset was gated for expression of CD45RA and CCR7, CD127 and PD-1.

### Statistical analysis

Was conducted using Prism 5 for MAC OSX. The Mann–Whitney *U* test was used for two sample comparisons. A Friedman test with Dunn's post-test comparison was used for non-parametric continuous data generated from repeated measures within individuals.

## Supporting Information

**Figure S1.** Differentiation status of CD4+ antigen-specific functional subsets.

**Figure S2.** Frequency of CD4+ cell subsets that expressed PD-1.

## References

[b1] WHO (2011). TB/HIV facts.

[b2] ECDC/WHO (2009).

[b3] Oni T, Burke R, Tsekela R, Bangani N, Seldon R, Gideon HP, Wood K, Wilkinson KA, Ottenhoff TH, Wilkinson RJ (2011). High prevalence of subclinical tuberculosis in HIV-1-infected persons without advanced immunodeficiency: implications for TB screening. Thorax.

[b4] Sonnenberg P, Glynn JR, Fielding K, Murray J, Godfrey-Faussett P, Shearer S (2005). How soon after infection with HIV does the risk of tuberculosis start to increase? A retrospective cohort study in South African gold miners. J. Infect. Dis.

[b5] Lawn SD, Harries AD, Williams BG, Chaisson RE, Losina E, De Cock KM, Wood R (2011). Antiretroviral therapy and the control of HIV-associated tuberculosis. Will ART do it. Int. J. Tuberc. Lung Dis.

[b6] Bronke C, Palmer NM, Jansen CA, Westerlaken GH, Polstra AM, Reiss P, Bakker M, Miedema F, Tesselaar K, van Baarle D (2005). Dynamics of cytomegalovirus (CMV)-specific T cells in HIV-1-infected individuals progressing to AIDS with CMV end-organ disease. J. Infect. Dis.

[b7] Petrara MR, Freguja R, Gianesin K, Zanchetta M, De Rossi A (2013). Epstein-Barr virus-driven lymphomagenesis in the context of human immunodeficiency virus type 1 infection. Front. Microbiol.

[b8] Engels EA, Pfeiffer RM, Landgren O, Moore RD (2010). Immunologic and virologic predictors of AIDS-related non-hodgkin lymphoma in the highly active antiretroviral therapy era. J. Acquir. Immune Defic. Syndr.

[b9] Owotade FJ, Patel M, Ralephenya TR, Vergotine G (2013). Oral Candida colonization in HIV-positive women: associated factors and changes following antiretroviral therapy. J. Med. Microbiol.

[b10] Nkuize M, De Wit S, Muls V, Arvanitakis M, Buset M (2010). Upper gastrointestinal endoscopic findings in the era of highly active antiretroviral therapy. HIV Med.

[b11] Miranda A, Morgan M, Jamal L, Laserson K, Barreira D, Silva G, Santos J, Wells C, Paine P, Garrett D (2007). Impact of antiretroviral therapy on the incidence of tuberculosis: the Brazilian experience, 1995–2001. PLoS ONE.

[b12] Lawn SD, Bekker LG, Wood R (2005). How effectively does HAART restore immune responses to *Mycobacterium tuberculosis*? Implications for tuberculosis control. AIDS.

[b13] Dunham RM, Cervasi B, Brenchley JM, Albrecht H, Weintrob A, Sumpter B, Engram J, Gordon S, Klatt NR, Frank I (2008). CD127 and, CD25 expression defines CD4+ T cell subsets that are differentially depleted during HIV infection. J. Immunol.

[b14] Lawn SD, Badri M, Wood R (2005). Tuberculosis among HIV-infected patients receiving HAART: long term incidence and risk factors in a South African cohort. AIDS.

[b15] Geldmacher C, Ngwenyama N, Schuetz A, Petrovas C, Reither K, Heeregrave EJ, Casazza JP, Ambrozak DR, Louder M, Ampofo W (2010). Preferential infection and depletion of *Mycobacterium tuberculosis*-specific CD4 T cells after HIV-1 infection. J. Exp. Med.

[b16] Hu H, Nau M, Ehrenberg P, Chenine AL, Macedo C, Zhou Y, Daye ZJ, Wei Z, Vahey M, Michael NL (2013). Distinct gene-expression profiles associated with the susceptibility of pathogen-specific CD4 T cells to HIV-1 infection. Blood.

[b17] Day CL, Mkhwanazi N, Reddy S, Mncube Z, van der Stok M, Klenerman P, Walker BD (2008). Detection of polyfunctional *Mycobacterium tuberculosis*-specific T cells and association with viral load in HIV-1-infected persons. J. Infect. Dis.

[b18] Harari A, Vallelian F, Meylan PR, Pantaleo G (2005). Functional heterogeneity of memory CD4 T cell responses in different conditions of antigen exposure and persistence. J. Immunol.

[b19] Harari A, Petitpierre S, Vallelian F, Pantaleo G (2004). Skewed representation of functionally distinct populations of virus-specific CD4 T cells in HIV-1-infected subjects with progressive disease: changes after antiretroviral therapy. Blood.

[b20] Boaz MJ, Waters A, Murad S, Easterbrook PJ, Vyakarnam A (2002). Presence of HIV-1 Gag-specific IFN-gamma + IL-2+ and CD28 + IL-2+ CD4 T cell responses is associated with nonprogression in HIV-1 infection. J. Immunol.

[b21] Casey R, Blumenkrantz D, Millington K, Montamat-Sicotte D, Kon OM, Wickremasinghe M, Bremang S, Magtoto M, Sridhar S, Connell D (2010). Enumeration of functional T-cell subsets by fluorescence-immunospot defines signatures of pathogen burden in tuberculosis. PLoS ONE.

[b22] Day CL, Abrahams DA, Lerumo L, Janse van Rensburg E, Stone L, O'Rie T, Pienaar B, de Kock M, Kaplan G, Mahomed H (2011). Functional capacity of *Mycobacterium tuberculosis*-specific T cell responses in humans is associated with mycobacterial load. J. Immunol.

[b23] Lalvani A, Millington KA (2008). T cells and tuberculosis: beyond interferon-gamma. J. Infect. Dis.

[b24] Lahey T, Sheth S, Matee M, Arbeit R, Horsburgh CR, Mtei L, Mackenzie T, Bakari M, Vuola JM, Pallangyo K (2010). Interferon gamma responses to mycobacterial antigens protect against subsequent HIV-associated tuberculosis. J. Infect. Dis.

[b25] Kalsdorf B, Scriba TJ, Wood K, Day CL, Dheda K, Dawson R, Hanekom WA, Lange C, Wilkinson RJ (2009). HIV-1 Infection impairs the bronchoalveolar T-Cell Response to Mycobacteria. Am. J. Respir. Crit. Care Med.

[b26] Chetoui N, Boisvert M, Gendron S, Aoudjit F (2010). Interleukin-7 promotes the survival of human CD4+ effector/memory T cells by up-regulating Bcl-2 proteins and activating the JAK/STAT signalling pathway. Immunology.

[b27] Schluns KS, Lefrancois L (2003). Cytokine control of memory T-cell development and survival. Nat. Rev. Immunol.

[b28] Appay V, Dunbar PR, Callan M, Klenerman P, Gillespie GM, Papagno L, Ogg GS, King A, Lechner F, Spina CA (2002). Memory CD8+ T cells vary in differentiation phenotype in different persistent virus infections. Nat. Med.

[b29] Riou C, Treurnicht F, Abrahams MR, Mlisana K, Liu MK, Goonetilleke N, Koup R, Roederer M, Abdool Karim S, de Bruyn G (2012). Increased memory differentiation is associated with decreased polyfunctionality for HIV but not for cytomegalovirus-specific CD8+ T cells. J. Immunol.

[b30] Libri V, Azevedo RI, Jackson SE, Di Mitri D, Lachmann R, Fuhrmann S, Vukmanovic-Stejic M, Yong K, Battistini L, Kern F (2011). Cytomegalovirus infection induces the accumulation of short-lived, multifunctional CD4+ CD45RA+ CD27+ T cells: the potential involvement of interleukin-7 in this process. Immunology.

[b31] Sallusto F, Geginat J, Lanzavecchia A (2004). Central memory and effector memory T cell subsets: function, generation, and maintenance. Annu. Rev. Immunol.

[b32] Pollock KM, Whitworth HS, Montamat-Sicotte DJ, Grass L, Cooke GS, Kapembwa MS, Kon OM, Sampson RD, Taylor GP, Lalvani A (2013). T-cell immunophenotyping distinguishes active from latent tuberculosis. J. Infect. Dis.

[b33] Freeman GJ, Wherry EJ, Ahmed R, Sharpe AH (2006). Reinvigorating exhausted HIV-specific T cells via PD-1-PD-1 ligand blockade. J. Exp. Med.

[b34] Porichis F, Kwon DS, Zupkosky J, Tighe DP, McMullen A, Brockman MA, Pavlik DF, Rodriguez-Garcia M, Pereyra F, Freeman GJ (2011). Responsiveness of HIV-specific CD4 T cells to PD-1 blockade. Blood.

[b35] Jurado JO, Alvarez IB, Pasquinelli V, Martinez GJ, Quiroga MF, Abbate E, Musella RM, Chuluyan HE, Garcia VE (2008). Programmed death (PD)-1:PD-ligand 1/PD-ligand 2 pathway inhibits T cell effector functions during human tuberculosis. J. Immunol.

[b36] Lazar-Molnar E, Chen B, Sweeney KA, Wang EJ, Liu W, Lin J, Porcelli SA, Almo SC, Nathenson SG, Jacobs WR (2010). Programmed death-1 (PD-1)-deficient mice are extraordinarily sensitive to tuberculosis. Proc. Natl. Acad. Sci. U. S. A.

[b37] Dosanjh DP, Hinks TS, Innes JA, Deeks JJ, Pasvol G, Hackforth S, Varia H, Millington KA, Gunatheesan R, Guyot-Revol V (2008). Improved diagnostic evaluation of suspected tuberculosis. Ann. Intern. Med.

[b38] Amyes E, Hatton C, Montamat-Sicotte D, Gudgeon N, Rickinson AB, McMichael AJ, Callan MF (2003). Characterization of the CD4+ T cell response to Epstein-Barr virus during primary and persistent infection. J. Exp. Med.

